# *ACTA1* gene regulation in livestock: A multidimensional review on muscle development, meat quality, and genetic applications

**DOI:** 10.14202/vetworld.2025.2520-2541

**Published:** 2025-08-30

**Authors:** Siti Rani Ayuti, Sangsu Shin, Eun Joong Kim, Mirni Lamid, Sunaryo Hadi Warsito, Mohammad Anam Al Arif, Widya Paramita Lokapirnasari, Zulfi Nur Amrina Rosyada, Aswin Rafif Khairullah, Muslim Akmal, Mudhita Zikkrullah Ritonga, Rimayanti Rimayanti, Mira Delima

**Affiliations:** 1 Doctoral Program of Veterinary Science, Faculty of Veterinary Medicine, Universitas Airlangga, Jl. Dr. Ir. H. Soekarno, Kampus C Mulyorejo, Surabaya 60115, East Java, Indonesia; 2 Laboratory of Biochemistry, Faculty of Veterinary Medicine, Universitas Syiah Kuala, Banda Aceh, Indonesia, Jl. Tgk. Hasan Krueng Kalee No.4, Kopelma Darussalam, Banda Aceh 23111, Aceh, Indonesia; 3 Department of Animal Science and Biotechnology, Kyungpook National University, Sangju 37224, Republic of Korea; 4 Division of Animal Husbandry, Faculty of Veterinary Medicine, Universitas Airlangga, Jl. Dr. Ir. H. Soekarno, Kampus C Mulyorejo, Surabaya 60115, East Java, Indonesia; 5 Research Center for Veterinary Science, National Research and Innovation Agency (BRIN), Jl. Raya Bogor Km. 46 Cibinong, Bogor 16911, West Java, Indonesia; 6 Laboratory of Histology, Faculty of Veterinary Medicine, Universitas Syiah Kuala, Banda Aceh, Indonesia, Jl. Tgk. Hasan Krueng Kalee No.4, Kopelma Darussalam, Banda Aceh 23111, Aceh, Indonesia; 7 Laboratory of Anatomy, Faculty of Veterinary Medicine, Universitas Syiah Kuala, Banda Aceh, Indonesia, Jl. Tgk. Hasan Krueng Kalee No.4, Kopelma Darussalam, Banda Aceh 23111, Aceh, Indonesia; 8 Division of Veterinary Reproduction, Faculty of Veterinary Medicine, Universitas Airlangga, Jl. Dr. Ir. H. Soekarno, Kampus C Mulyorejo, Surabaya 60115, East Java, Indonesia; 9 Department of Animal Husbandry, Faculty of Agriculture, Universitas Syiah Kuala, Banda Aceh, Indonesia, Jl. Tgk. Hasan Krueng Kalee No.4, Kopelma Darussalam, Banda Aceh 23111, Aceh, Indonesia

**Keywords:** ACTA1, genetic diversity, livestock, meat quality, skeletal muscle

## Abstract

The skeletal muscle α-actin gene (ACTA1) plays a pivotal role in muscle contraction, structural integrity, and overall development of skeletal muscle tissue in livestock. This review explores the complex regulatory mechanisms of ACTA1 expression and its direct impact on meat quality, animal performance, and production efficiency. Nutritional inputs, environmental stressors, hormonal signaling, and genetic factors collectively influence ACTA1 activity at the transcriptional, translational, and epigenetic levels. High-protein diets rich in branched-chain amino acids, particularly leucine, stimulate the mechanistic target of rapamycin pathway and enhance ACTA1-mediated muscle growth. Similarly, micronutrients such as zinc and selenium function as antioxidants, stabilizing ACTA1 expression under oxidative stress conditions. The review also delves into the role of ACTA1 polymorphisms in modulating muscle fiber type composition, particularly the balance between type I and type II fibers, which significantly affects meat tenderness, fat content, and endurance capacity. Genome-wide association studies, marker-assisted selection (MAS), and clustered regularly interspaced short palindromic repeats-associated protein 9-based genome editing provide promising avenues for optimizing ACTA1 expression in livestock breeding programs. Moreover, ACTA1 dysregulation or mutation is linked to several congenital myopathies, underscoring its diagnostic and therapeutic relevance in veterinary pathology. Biotechnological interventions targeting ACTA1 expression present immense potential for improving muscle mass, carcass traits, and feed efficiency, thereby supporting global food security. Future strategies combining nutrigenomics, precision livestock farming, and artificial intelligence could enable tailored breeding and management approaches for sustainable meat production. Ethical and environmental considerations will be critical as gene editing technologies move toward wider application. In summary, ACTA1 represents a cornerstone of muscle physiology in livestock, and its integrative regulation across nutrition, genetics, and environment offers vast potential for advancing meat science, animal health, and agricultural productivity.

## INTRODUCTION

Precision farming and nutrigenomics technologies offer innovative strategies to enhance α-actin (ACTA1) expression in skeletal muscle, thereby improving meat quality [1]. *RNA-seq-*based gene expression profiling enables the identification of ACTA1 expression patterns under diverse environmental and dietary conditions, supporting the development of tailored nutritional strategies [2]. In addition, sensor technologies and artificial intelligence allow for early detection of physiological stressors in livestock that may impair ACTA1 expression and hinder muscle development [3]. The integration of precision nutrition, environmental management, and advanced monitoring systems can significantly improve both livestock productivity and meat quality [4, 5].

Nutritional regulation plays a critical role in maintaining optimal ACTA1 expression, which directly influences meat quality [6]. Adequate nutrition ensures a consistent supply of amino acids and macronutrients essential for muscle protein synthesis [7]. High-quality proteins containing essential amino acids–particularly valine, leucine, and isoleucine–activate the mechanistic target of rapamycin (mTOR) pathway, thereby enhancing ACTA1 synthesis [8, 9]. A balanced amino acid composition in animal feed supports muscle growth, prevents degradation, and helps maintain meat quality [10, 11]. Enhancing ACTA1 expression and muscle development depends on diets rich in complete proteins and the appropriate ratio of essential amino acids [12, 13].

ACTA1 expression is closely tied to muscle fiber formation. Adequate dietary intake of protein and essential amino acids supports both muscle protein synthesis and *ACTA1* gene activation [14–16]. Activation of the mTOR signaling pathway through protein intake promotes muscle growth [17]. Carbohydrates and omega-3 fatty acids may also regulate gene expression, contributing to effective muscle development [18, 19]. Proper nutritional control, especially during growth phases, is crucial for maintaining ACTA1 expression and improving meat quality [20]. High-protein feeds and hydrolyzed amino acid supplements have been shown to enhance ACTA1 synthesis and accelerate muscle growth [21–26]. Specialized high-protein formulations can further drive ACTA1 expression, leading to superior meat quality and market value [27, 28].

Genomic selection offers a modern approach to increasing the expression of performance-related genes like ACTA1 [29, 30]. This technique enables researchers to analyze the entire genome and identify variants associated with higher ACTA1 expression levels [22, 24]. Breeding animals with optimal genetic potential for muscle development and meat quality becomes more efficient using such genomic insights [16]. Compared to traditional selection methods, genomic selection accelerates breeding cycles and yields faster results [14, 16, 18]. Gene therapy and genomic selection combined hold strong potential to improve ACTA1 expression and overall livestock productivity [6, 9]. Advances in gene editing technologies such as clustered regularly interspaced short palindromic repeats-associated protein 9 (CRISPR/Cas9) have made it feasible to directly target ACTA1, enhancing growth efficiency and muscle quality [22, 23]. When genomic selection is paired with improved husbandry and feed management, the benefits to meat production are amplified [18, 20, 27].

Despite substantial advances in livestock genomics, nutrition, and biotechnology, the mechanistic understanding of ACTA1 regulation and its integration across dietary, genetic, and environmental contexts remains fragmented. Most current studies investigate ACTA1 in relation to congenital myopathies or human muscle physiology, with limited emphasis on its functional modulation in livestock for improving production traits. In addition, while individual elements–such as protein supplementation, mTOR pathway activation, or gene editing techniques–have been studied in isolation, a comprehensive model linking nutritional regulation, transcriptional control, and biotechnological manipulation of ACTA1 in meat-producing animals is lacking. There is also a need for comparative evaluation of ACTA1 expression among different livestock species, breeds, and production systems, especially under varying physiological stress and growth conditions. Furthermore, although CRISPR/Cas9 and MAS show promise in livestock gene targeting, empirical studies applying these tools specifically to optimize ACTA1 expression for muscle development and meat quality enhancement are still sparse.

This review aims to synthesize current knowledge on the molecular regulation of ACTA1 in skeletal muscle, with a particular focus on its application to meat quality improvement, muscle fiber differentiation, and performance enhancement in livestock. It explores how nutritional strategies, hormonal pathways, environmental modulation, and genomic technologies interact to influence ACTA1 expression. The study further examines the role of ACTA1 polymorphisms, gene expression dynamics, and gene-editing interventions in shaping muscle structure and function. By integrating insights from nutrigenomics, precision farming, and genome engineering, this review seeks to highlight promising biotechnological approaches and identify actionable strategies for optimizing ACTA1 expression to support sustainable and high-quality meat production across species.

## ROLE OF ACTA1 IN MUSCLE CONTRACTION AND LIVESTOCK PERFORMANCE

ACTA1 encodes the skeletal muscle protein α-actin, which is essential for muscular contraction [31]. The optimal function of ACTA1 directly influences livestock performance by enhancing muscle contractility [17, 22]. Animals with well-developed muscles exhibit improved mobility, feed efficiency, and overall growth performance [7, 31]. Enhancing livestock performance is crucial for meeting the increasing global demand for high-quality animal protein [32]. In addition to improving animal performance, ACTA1 expression contributes to superior meat quality [24]. Optimal α-actin function helps maintain meat structure and texture, resulting in improved tenderness and chewiness, which is desirable to consumers [10, 19].

## ACTA1 IN GENETIC REGULATION OF MEAT YIELD AND QUALITY

Genetic research in livestock aims to optimize ACTA1 expression to enhance muscle yield and carcass traits [33]. ACTA1 plays a central role in the genetic regulation of skeletal muscle function in livestock [34]. Understanding ACTA1 allows for genetic selection strategies that improve muscle traits and the genetic quality of livestock [12]. ACTA1 is a potential target for genetic engineering to enhance skeletal muscle growth in animals [17]. Such interventions can result in faster-growing animals with improved feed conversion and superior meat quality [27, 29].

Incorporating ACTA1-focused genetic strategies can increase livestock production system productivity and meat yield [16, 20]. ACTA1-targeted breeding supports global efforts to increase the supply of high-quality animal protein [9, 23]. ACTA1 significantly affects carcass quality and overall meat characteristics [12]. The functional characteristics of ACTA1 offer numerous opportunities for improving meat production in the livestock sector [3]. Genetic engineering and selective breeding targeting ACTA1 can enhance productivity and support the production of premium meat products that meet global market expectations [7, 35]. Carcass weight, a key indicator of production success, is largely determined by well-developed, healthy skeletal muscle [11]. Therefore, managing ACTA1 expression through genetic selection is a vital strategy in modern animal breeding programs [36].

## IMPACT OF ACTA1 MUTATIONS ON MUSCLE FUNCTION AND PRODUCTIVITY

Environmental and physiological conditions can significantly influence the growth, performance, and carcass characteristics of livestock [12]. Early identification of ACTA1 variants is essential to mitigate the potential negative impacts on animal productivity [6]. Mutations in the *ACTA1* gene can severely impair muscle function, especially in livestock, by hindering muscle development [19]. Such mutations may disrupt the structure and function of α-actin, compromising the muscle’s ability to contract efficiently [11, 22].

ACTA1 mutations negatively affect muscle development and may decrease growth rates and efficiency in livestock [7]. Insufficient muscle development due to ACTA1 dysfunction hinders the achievement of optimal body weight in animals [33]. Animals with ACTA1 mutations tend to exhibit reduced muscle mass, resulting in poorer carcass quality and suboptimal meat yield [14]. These mutations are also associated with structural muscle abnormalities, such as myopathies and generalized muscle weakness [8]. ACTA1-related myopathies often impair locomotion, adversely affecting metabolism and activity levels in livestock [37]. These impairments reduce feed efficiency and endurance, ultimately decreasing overall productivity [11].

Genetic selection for favorable ACTA1 variants is a key strategy for counteracting the negative effects of gene mutations on muscle development [6, 11, 13]. Advanced genetic engineering techniques offer the potential to correct deleterious mutations, promoting stronger and healthier musculature development [33]. A deeper understanding of the role of ACTA1 in muscle biology can support efforts to improve livestock performance and meat production [27].

## BIOTECHNOLOGICAL POTENTIAL OF ACTA1 IN LIVESTOCK IMPROVEMENT

While ACTA1 is essential for muscle contraction, it also holds significant potential for biotechnology and genetic improvement applications [37]. ACTA1 encodes the α-actin protein, a critical component of thin filaments within skeletal muscle sarcomeres that facilitates effective muscle contraction [25]. ACTA1 is under investigation for its potential to improve muscle quality and enhance disease resilience in livestock through biotechnological innovation [38].

Understanding the regulatory mechanisms and expression patterns of ACTA1 enables researchers to identify factors that influence muscle function in livestock [12]. This knowledge can inform genetic selection strategies targeting individuals with optimal ACTA1 expression, thereby improving the success of breeding programs [7, 32]. Such approaches can accelerate growth rates, increase muscle strength and efficiency, and improve overall muscular performance in animals [15, 22]. The increasing consumer demand for premium meat underscores the need to enhance livestock performance and production efficiency [17, 28]. The livestock industry can meet rising market demands by adopting genetic and biotechnological methods to improve productivity and product quality [38].

## STRUCTURAL ROLE OF ACTA1 IN MUSCLE FIBER ARCHITECTURE

Like other actins, ACTA1 contains a highly conserved ATP-binding domain responsible for filament polymerization [33]. It interacts with cytoskeletal proteins, such as myosin, to form actin filaments, which are essential components of the contractile apparatus [26]. Actin collaborates with tropomyosin and regulatory proteins through the sliding filament mechanism to facilitate muscle contraction [12]. These interactions are vital for maintaining filament integrity and mechanical resilience during muscle contraction [29].

The polymerization of G-actin into F-actin is a fundamental mechanism underpinning cytoskeletal integrity and muscle contractility [39]. Actin monomers assemble into helical filaments through longitudinal and lateral interactions, forming stable contractile structures [7]. Specific ACTA1 mutations can weaken inter-monomer bonding, resulting in unstable or malformed actin filaments [40]. In actin-related myopathies, such abnormalities compromise sarcomere structure, reduce contractile force, and contribute to muscle weakness [23].

## MOLECULAR INSIGHTS INTO ACTA1 MUTATIONS AND MYOPATHIES

Model systems, including skeletal muscle cell cultures and animal models, have clarified how ACTA1 mutations affect actin filament dynamics and disrupt muscle function [19]. ACTA1 mutations are associated with several inherited myopathies characterized by structural muscle defects and progressive weakness [25]. The severity of ACTA1-related myopathies varies depending on the specific mutation and its impact on filament organization [41]. Common mutations include substitutions, deletions, or insertions that impair protein structure and function [42]. Induced pluripotent stem cell-derived muscle cells and transgenic mouse models enable detailed examination of these molecular consequences [16, 22].

Advanced techniques–such as molecular dynamics simulations, live-cell imaging, and cryo-electron microscopy–have enhanced understanding of ACTA1 mutations at the molecular level [11, 28]. These tools allow real-time monitoring of structural alterations and high-resolution visualization of actin filament dynamics [30].

## ACTA1 AND SARCOMERE REGULATION THROUGH PROTEIN INTERACTIONS

Beyond actin-actin interactions, ACTA1 interacts with structural proteins such as tropomyosin, troponin, and myosin, all of which are critical to sarcomere function [15]. Tropomyosin regulates myosin-binding sites along F-actin, ensuring coordinated muscle contraction [10]. These protein interactions provide key insights into the pathophysiology of actin-related myopathies [6].

Recent advances in structural biology and molecular modeling have enabled the design of targeted therapies that aim to correct ACTA1 mutations. These include small molecules that stabilize F-actin and gene editing approaches aimed at restoring normal muscle function [2, 9, 19].

## TRANSCRIPTIONAL REGULATION OF ACTA1 EXPRESSION

The ACTA1 promoter contains key regulatory elements that control its muscle-specific expression. These include binding sites for transcription factors such as MyoD, myogenin, myocyte enhancer factor 2 (MEF2), and serum response factor (SRF), which regulate gene activation during myogenesis [13, 19, 23, 31]. These transcriptional controls ensure that ACTA1 is expressed in developing and mature muscle cells while preventing expression in non-muscle tissues [40].

Myogenic regulatory factors (MRFs) such as MyoD and myogenin bind to E-box motifs in the ACTA1 promoter to drive expression during muscle development [20, 37, 42]. These transcription factors initiate ACTA1 transcription and are upregulated during myoblast differentiation [21]. MEF2 and SRF respond to mechanical stimuli and signaling pathways to sustain ACTA1 expression in mature fibers, supporting continuous α-actin synthesis [20].

## EPIGENETIC AND SIGNALING DISRUPTION OF ACTA1 TRANSCRIPTION

ACTA1 expression can be impaired by mutations or epigenetic alterations that disrupt transcriptional regulation, contributing to muscle weakness and degenerative myopathies [19]. Congenital myopathies may result from decreased or abnormal α-actin production due to mutations in the ACTA1 promoter or chromatin modifications [8, 11].

Alterations in promoter regions that hinder transcription factor binding can reduce ACTA1 transcription and disrupt sarcomere assembly [15, 25]. Dysregulation in signaling pathways–such as those associated with muscle atrophy or oxidative stress–can suppress ACTA1 promoter activity, exacerbating degeneration in conditions such as nemaline myopathy [37, 43].

## EMERGING THERAPEUTIC STRATEGIES TARGETING ACTA1

Given the critical role of ACTA1 in muscle-specific expression and contractility, several therapeutic strategies are under investigation to restore its function [13]. CRISPR-based correction of promoter mutations and epigenetic therapies, such as histone deacetylase inhibitors, shows promise for treating ACTA1-related disorders [19, 44].

ACTA1 mutations are implicated in various hereditary muscle diseases, including nemaline myopathy, actin myopathy, and intranuclear rod myopathy [5]. These conditions often manifest as hypotonia, muscle weakness, and, in severe cases, respiratory failure due to impaired diaphragm contraction [30]. Pathogenic mutations can disrupt actin polymerization or cause protein misfolding, leading to toxic aggregate accumulation in muscle fibers [22, 38].

Recent advances in gene therapy and molecular biology offer hope for these conditions. Current therapeutic investigations include gene replacement, RNA-based interventions, and small molecules that stabilize actin filaments to restore muscle function [12, 19, 45].

## ACTA1 IN MUSCLE HOMEOSTASIS AND PROTEIN TURNOVER

Muscle homeostasis relies on a finely tuned balance between protein synthesis and degradation, growth factor activity, and satellite cell-mediated regeneration [15]. These biological processes collectively modulate ACTA1 expression in response to hormonal cues, mechanical stress, and the developmental stage of muscle fibers [19]. As a core component of thin filaments in skeletal muscle, ACTA1 plays a pivotal role in maintaining sarcomere integrity and facilitating efficient muscle contraction [32]. Sustaining this dynamic balance is vital for optimal muscle function during development, physical activity, and disease conditions [40].

## REGULATORY PATHWAYS MODULATING ACTA1 EXPRESSION

ACTA1 synthesis is regulated by nutrient- and activity-sensitive pathways such as mTOR and AMP-activated protein kinase, which respond to nutritional availability and exercise stimuli [29]. Disruptions in these pathways may result in muscle hypertrophy, atrophy, or weakness, depending on the nature of the imbalance [35]. Mechanically induced muscle adaptation involves critical pathways including insulin-like growth factor 1 (IGF-1)/PI3K/Akt and Hippo-YAP, both of which directly influence ACTA1 transcription and muscle remodeling under load [39]. Moreover, repetitive physical activity increases ACTA1 expression, reinforcing actin filament assembly and enhancing contractile efficiency [10, 24]. Mechanotransduction mechanisms, particularly those mediated by FAK (focal adhesion kinase) and Hippo-YAP, also boost ACTA1 transcription, increasing the muscle’s resistance to mechanical stress [18].

## ACTA1 AND CELLULAR ADAPTATION TO OXIDATIVE AND METABOLIC STRESS

Beyond mechanical signaling, ACTA1 plays a crucial role in adaptation to oxidative and metabolic stress [22]. Under pathological conditions such as muscular dystrophy, reactive oxygen species can damage ACTA1 and other key contractile proteins, compromising muscle function [37]. To maintain proteostasis, muscle cells activate autophagy and the ubiquitin-proteasome system, which degrade damaged proteins and prevent the buildup of toxic aggregates [31, 41]. Disruptions in ACTA1 integrity can lead to pathological conditions such as nemaline myopathy, characterized by the accumulation of abnormal actin filaments and progressive muscle degeneration [46].

## IMPLICATIONS OF ACTA1 DYSFUNCTION IN MUSCLE REGENERATION

In addition to maintaining muscle structure, ACTA1 is essential for muscle regeneration. Satellite cell activation, a prerequisite for muscle repair, depends on a structurally intact sarcomere framework supported by proper ACTA1 function [37]. Any disruptions in ACTA1 expression or protein stability can delay regenerative processes and increase the risk of fibrosis or muscle atrophy [18]. These dysfunctions may arise either from genetic mutations or dysregulated transcriptional control [37].

## THERAPEUTIC INTERVENTIONS TARGETING ACTA1 DYSREGULATION

A comprehensive understanding of ACTA1’s role in muscle homeostasis and stress adaptation may facilitate the development of targeted therapies for muscle disorders [47]. Current approaches include gene-editing techniques such as CRISPR/Cas9, small molecules that stabilize actin filaments, and epigenetic modulators designed to restore proper ACTA1 expression [3, 12, 25]. In addition, nutritional therapies aimed at enhancing actin filament stability offer a non-invasive strategy to improve muscle resilience in individuals with ACTA1 dysfunction [25].

## NUTRITIONAL AND GENOMIC STRATEGIES FOR SUPPORTING ACTA1 FUNCTION

Dietary interventions that promote ACTA1 expression are increasingly recognized as effective methods for sustaining muscle health [29]. These strategies, when combined with pharmaceutical agents and genomic tools, hold promise for managing myopathies linked to ACTA1 mutations or regulatory defects [18]. Overall, enhancing ACTA1 expression and stability can improve muscle endurance, regenerative capacity, and resistance to physiological stressors, contributing to better muscle function and meat quality in livestock.

## ACTA1 AS A STRUCTURAL DETERMINANT OF MEAT QUALITY

ACTA1 is a major component of thin filaments in muscle fibers, playing a vital role in determining meat structure [14]. Its expression influences the density, texture, and contractile strength of muscle fibers–critical attributes in evaluating meat quality. The interaction of ACTA1 with signaling pathways that regulate muscle growth, differentiation, and maintenance significantly impacts meat development and consumer-preferred characteristics [19].

## GROWTH FACTOR REGULATION OF ACTA1 EXPRESSION

The stability and expression of ACTA1 are tightly regulated by key growth factors such as IGF-1, myostatin, and transforming growth factor-beta (TGF-β), all of which influence muscle mass and meat texture [22, 47, 48]. Enhancing meat quality through breeding, nutrition, or gene editing requires a mechanistic understanding of how ACTA1 functions within these regulatory frameworks [29].

## THE IGF-1/PI3K/AKT/MTOR AXIS AND ACTA1 SYNTHESIS

The IGF-1/PI3K/Akt pathway is a principal driver of ACTA1 expression and muscle hypertrophy [13]. IGF-1 activates PI3K and Akt, which in turn stimulate protein synthesis and suppress muscle degradation through the mTOR signaling cascade [26]. mTOR activation enhances the translation of contractile proteins, including ACTA1, thereby increasing muscle accretion and improving meat texture [32]. Nutritional supplementation with mTOR-activating agents such as leucine can further promote thin filament formation in sarcomeres and meat quality [49].

Conversely, mTOR suppression, due to disease, nutrient deficiency, or stress, can reduce ACTA1 synthesis, leading to muscle atrophy and inferior meat quality [30]. Therefore, both pharmacological and dietary modulation of the mTOR pathway present viable strategies for improving muscle growth and meat composition [41].

## WNT/Β-CATENIN PATHWAY AND MYOBLAST DIFFERENTIATION

**Figure 1:**
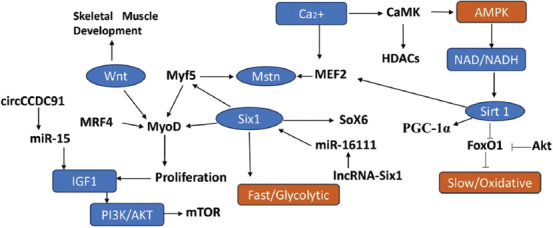
Network diagram of an animal regulating muscle fiber [Source: The figure was prepared by Siti Rani Ayuti].

In addition to mTOR, the Wnt/β-catenin signaling pathway plays a vital role in regulating ACTA1 during muscle development [42]. β-catenin functions as a key transcription factor that initiates the expression of muscle-specific genes, including ACTA1, and supports the differentiation of myoblasts into mature muscle fibers [12]. Selective breeding programs or epigenetic therapies that target the Wnt/ACTA1 axis may offer promising avenues for improving meat production efficiency [37]. Figure 1 is an illustration of a network diagram of an animal’s regulating muscle fiber.

## MYOSTATIN AS A NEGATIVE REGULATOR OF ACTA1

Myostatin, a TGF-β family member, is a well-known negative regulator of muscle growth. It inhibits ACTA1 synthesis by activating the Smad2/3 pathway and antagonizing the IGF-1/PI3K/Akt signaling cascade [48]. As a result, myostatin suppresses muscle proliferation and differentiation, contributing to reduced muscle mass and meat quality [37].

However, myostatin mutations or targeted inhibition have been shown to dramatically increase muscle mass and improve the protein content and fiber density of meat in multiple livestock species [18, 21, 22]. These enhancements can be achieved using *CRISPR/Cas9* gene editing, antibody-based therapies, or traditional genetic selection without compromising meat quality [24].

## HORMONAL AND GROWTH FACTOR CROSSTALK INFLUENCING ACTA1

In addition to myostatin and IGF-1, other growth factors–such as fibroblast growth factor, hepatocyte growth factor, and epidermal growth factor–also play roles in regulating ACTA1 expression [11, 50]. These factors influence muscle repair, regeneration, and adaptation to stress, contributing to final meat quality.

The balance between muscle protein synthesis and degradation, influenced by these hormonal signals and signaling cascades, ultimately determines the composition, texture, and nutritional value of the resulting meat product [36].

## BIOTECHNOLOGICAL MANIPULATION OF ACTA1 AND MYOSTATIN FOR MEAT ENHANCEMENT

Given their complementary roles, the co-regulation of ACTA1 and myostatin is a focal point for modern biotechnological strategies aimed at improving meat production [11, 48]. Through genome editing and targeted breeding, ACTA1 expression can be optimized while myostatin activity is selectively inhibited, resulting in livestock with larger muscle fibers, enhanced protein deposition, and higher-quality meat.

## INTEGRATING NUTRIGENOMICS INTO LIVESTOCK NUTRITION

Integrating nutrigenomics techniques into feed formulation offers a promising strategy to identify optimal nutrient combinations that enhance ACTA1 expression and promote efficient muscle growth [49]. Modern farm management technologies based on precision formulation can optimize the feeding environment and ensure animal welfare by monitoring real-time physiological responses [50]. Implementing scientifically backed feeding and husbandry protocols enables the livestock sector to produce higher-quality, healthier, and more economically valuable meat [51]. ACTA1, being essential for sarcomere structure and muscle contractility, is directly affected by nutritional status and environmental inputs [12].

## PROTEIN AND AMINO ACID REQUIREMENTS FOR ACTA1 SYNTHESIS

A balance of nutritional pillars, including high-quality protein, essential amino acids, micronutrients, environmental control, and physical activity, is vital for improving meat quality and supporting ACTA1 expression [51]. Among amino acids, branched-chain amino acids–leucine, isoleucine, and valine, are particularly crucial for ACTA1 production [52]. Leucine, in particular, activates the mTOR pathway, a central regulator of muscle protein synthesis and ACTA1 expression [22].

In addition, amino acids, such as glutamine and arginine support myoblast proliferation and differentiation, enhancing muscle fiber formation and ACTA1 upregulation [53]. A well-balanced protein-to-amino acid ratio in the feed formulation is necessary to optimize ACTA1 synthesis and ensure efficient muscle building [18].

## MICRONUTRIENTS SUPPORTING ACTA1 STABILITY

In addition to proteins and amino acids, micronutrients such as zinc, Vitamin D, and selenium also influence ACTA1 expression and muscle development [54]. Selenium and zinc act as antioxidants that protect ACTA1 and other structural proteins from oxidative stress and free radical-induced degradation [55]. Deficiencies in these micronutrients may disrupt ACTA1 expression, leading to muscle weakness and inferior meat quality [33]. Proper supplementation with essential micronutrients remains an effective strategy to support both muscle function and meat composition [29].

## ENVIRONMENTAL STRESS AND NUTRITIONAL SYNERGY

High levels of environmental stress, such as overcrowding, heat stress, or poor ventilation, can suppress ACTA1 expression and contribute to muscle degeneration and reduced meat quality [13]. Therefore, effective environmental management is critical to maintain ACTA1 activity and support optimal muscle development [56]. Nutritional strategies must work in tandem with environmental controls to mitigate stress-related gene downregulation and safeguard meat quality outcomes.

## OPTIMIZING FEED COMPOSITION AND MAINTENANCE FOR ACTA1 EXPRESSION

A precise balance of proteins and amino acids is essential for muscle fiber growth and regeneration, which is tightly linked to ACTA1 expression [24]. For example, glycine has been shown to activate the mTOR pathway, enhancing ACTA1 and general muscle protein synthesis [15]. The ideal combination of animal and plant protein sources in feed improves protein metabolism and promotes the development of healthier and more robust muscle fibers [17]. This results in superior meat texture and nutritional value [51].

Effective maintenance practices, including proper housing, movement space, and animal welfare considerations, further contribute to optimal ACTA1 expression and muscle development [8, 37]. A supportive living environment ensures that animals can express their full genetic potential for muscle growth when combined with tailored nutritional support [22].

## PRECISION FEEDING AND TECHNOLOGICAL INTEGRATION

Developing adaptive feed formulation systems that emphasize phase-specific nutrient requirements can help maintain ACTA1 expression across various growth stages [3]. Precision feeding, when combined with rigorous environmental monitoring, ensures that animals receive appropriate nutrition aligned with their physiological needs. This synergy between nutrition and management enhances meat production efficiency, product quality, and market value [29, 51, 56].

By leveraging the right blend of nutritional science, farm technology, and animal welfare, the livestock industry can unlock new levels of productivity while ensuring the biological sustainability of ACTA1 expression and muscle development.

## ACTA1 AS A KEY DRIVER OF MUSCLE CONTRACTION AND ENDURANCE

ACTA1 is a critical component of muscle thin filaments, playing a central role in facilitating contractile activity in muscle tissue [57]. Optimal ACTA1 expression enhances muscle performance and contraction endurance across various livestock species, supporting increased muscle mass and ultimately improving meat yield [58]. Cattle with higher ACTA1 levels typically produce denser meat with superior texture and reduced intramuscular fat, leading to greater commercial value [22, 58]. In pigs and poultry, optimized ACTA1 expression through genetic selection and nutrition contributes to muscle fibers with greater fatigue resistance and more refined texture [59, 60].

## MOLECULAR SIGNALING AND HUSBANDRY INFLUENCES ON ACTA1 FUNCTION

The enhancement of muscle performance by ACTA1 is mediated through its interactions with multiple molecular pathways involved in growth and endurance regulation [36]. While genetics and diet are major contributors, husbandry practices also significantly influence ACTA1 activity. Livestock raised under pasture-based or free-range systems generally exhibit improved muscle mass and contractility compared to animals housed in confined pens [61]. Pigs allowed mobility and structured exercise develop better muscle tone and meat texture [62]. Providing adequate space for locomotion and natural movement stimulates ACTA1 expression, improving muscle strength and endurance [63].

## SPECIES-SPECIFIC STRATEGIES FOR ACTA1 OPTIMIZATION

Although ACTA1 plays a universal role in muscle development, species-specific responses to genetic, nutritional, and environmental factors must be accounted for [13]. In beef cattle breeds, higher ACTA1 levels are genetically associated with larger muscle volume and higher protein content in meat [61]. For chickens, where muscle turnover is rapid, a feed rich in high-quality protein and amino acids is essential to support ACTA1-driven muscle performance [64]. In pigs, muscle size and contractility are closely tied to ACTA1-related genetic determinants, emphasizing the need for targeted nutritional and genetic strategies [65, 66].

## ACTA1 EXPRESSION AND ITS IMPACT ON MEAT YIELD AND TEXTURE

ACTA1’s structural role in sarcomeres contributes to muscle fiber density and meat tenderness [37]. Muscles with elevated ACTA1 levels develop stronger and denser fibers, resulting in premium meat texture and flavor [22]. In cattle, enhanced ACTA1 expression contributes to improved feed efficiency, reduced fat accumulation, and accelerated lean muscle growth [61]. In poultry, ACTA1 enhancement leads to greater fiber density and fatigue resistance, improving meat softness and market desirability [64]. Understanding the regulatory mechanisms of ACTA1 supports the design of precision breeding and nutrition protocols for superior meat production [33].

## GENETIC AND HUSBANDRY INTERVENTIONS TO ENHANCE ACTA1 EXPRESSION

Optimizing ACTA1 expression involves a synergistic approach combining genomic selection and evidence-based husbandry [25]. Breeders can use genomic tools to identify individuals with naturally high ACTA1 expression linked to enhanced muscle functionality [40]. Husbandry techniques that promote mechanotransduction, such as allowing free movement or exercise, can stimulate muscle growth and further activate ACTA1 pathways [23]. For instance, calves raised with greater mobility opportunities develop better muscle tone and produce leaner, high-quality meat [67]. Integrating genetic interventions with supportive rearing practices ensures maximal muscle quality and endurance [47].

## TOWARD SUSTAINABLE MEAT QUALITY THROUGH ACTA1 REGULATION

Further research is needed to clarify the interaction between genetics, nutrition, and the environment in modulating ACTA1 expression and enhancing muscle endurance [67]. Emerging studies suggest that combining balanced nutrition with optimized husbandry leads to more consistent upregulation of ACTA1, improving muscle strength and meat productivity [47]. Nutrigenomics tools offer pathways to design species-specific feed formulations tailored to ACTA1 regulatory profiles [68]. By leveraging molecular biology advancements and technological innovations, livestock producers can sustainably elevate meat quality while improving muscle resilience and endurance [32, 69].

## HARNESSING BIOTECHNOLOGY TO ENHANCE ACTA1 EXPRESSION

Biotechnological advancements offer promising avenues for optimizing ACTA1 expression to improve muscle growth and meat quality in livestock [40]. Through genetic engineering techniques, researchers can enhance muscle fiber size and strength by targeting the *ACTA1* gene or its regulatory pathways [54]. This can lead to livestock with increased muscle mass, directly translating into higher yields of premium-quality meat [21]. Among these technologies, CRISPR-Cas9 has revolutionized gene editing, allowing precise modifications to correct genetic defects that hinder ACTA1 expression, thereby improving a long-term muscular function in animals [70].

## GENOMIC SELECTION AND BREEDING STRATEGIES

**Figure 2:**
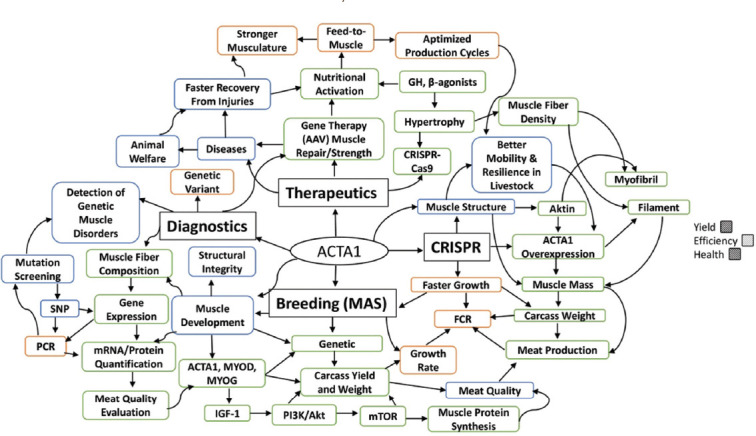
Applications of ACTA1 in Livestock Biotechnology, Breeding, clustered regularly interspaced short palindromic repeats, Diagnostics, and Therapeutics [Source: The figure was prepared by Siti Rani Ayuti].

In addition to direct gene editing, genomic selection tools enable breeders to identify animals with naturally higher ACTA1 expression, contributing to better muscle traits such as texture, softness, and lower fat content [21, 36]. By leveraging genome sequencing and mapping, researchers can pinpoint favorable ACTA1-related genetic variants, supporting targeted breeding programs that prioritize muscular performance and meat quality [61, 65, 71]. Incorporating ACTA1 expression into selection criteria enhances the genetic potential of future generations and increases the efficiency and productivity of livestock systems [67, 72]. Figure 2 is an illustration of applications of ACTA1 in livestock biotechnology, breeding, CRISPR, diagnostics, and therapeutics.

## CHALLENGES AND THE NEED FOR MULTIDISCIPLINARY INTEGRATION

Despite its promise, ACTA1-focused biotechnology must be applied cautiously to avoid ecological imbalance or unintended physiological consequences [25, 33, 73]. Successful implementation requires a multidisciplinary approach, integrating genetics, nutritional science, and livestock management [17]. Nutritional inputs, including micronutrients and essential amino acids, play a critical role in supporting ACTA1 expression at the molecular level [11, 37]. Feeding strategies that incorporate these components can further amplify the benefits of biotechnological interventions. Simultaneously, husbandry practices that encourage physical activity reinforce muscle protein synthesis and overall muscle health [69, 74].

## IMPACTS ON MEAT COMPOSITION AND MARKET QUALITY

Genetic manipulation of ACTA1 has the potential to dramatically enhance the composition of livestock meat, producing animals with denser muscles, reduced intramuscular fat, and improved meat texture [12, 64, 75]. These changes meet consumer preferences in a global market increasingly focused on high-protein, low-fat meat products [42, 76]. In addition to quantity, the overall quality and economic value of meat products are expected to improve through ACTA1-targeted biotechnology [77, 78]. Strategic integration of these tools can reduce production waste, optimize feed conversion, and partially meet growing global demand for high-quality animal protein [79, 80].

## FUTURE OUTLOOK AND SUSTAINABLE APPLICATIONS

The use of biotechnology to enhance ACTA1 expression is poised for continued expansion, with innovations in CRISPR-Cas9 techniques enabling increasingly precise and efficient edits to livestock genomes [70, 71]. Integrating big data analytics and sensor technologies into animal health and metabolism monitoring systems will yield deeper insights into the lifespan effects of ACTA1 modifications [75]. These developments will not only improve the efficiency and sustainability of meat production but also contribute to global food security by ensuring a consistent supply of nutritious, high-quality meat [78, 80].

## ACTA1 MUTATIONS AND CONGENITAL MYOPATHIES

Mutations in the *ACTA1* gene, which encodes skeletal muscle α-actin, are frequently associated with congenital myopathies; a group of inherited muscle disorders characterized by progressive muscle weakness, stiffness, and impaired mobility [70, 81, 82]. These disorders are categorized into two main types: Actin myopathy and myofibrillar myopathy [83]. Actin myopathy is typically marked by gradual muscle deterioration, whereas myofibrillar myopathy presents more severe muscle structural abnormalities due to protein aggregation in muscle cells [84].

The pathological basis lies in mutations that disrupt the structure or function of α-actin, affecting its polymerization and interaction with myosin, and thereby impairing sarcomere integrity [85–88]. These disruptions result in weak, malformed, or misfolded actin filaments, reducing contractile efficiency and increasing muscle fatigue.

## IMPLICATIONS OF ACTA1 MUTATIONS IN POULTRY HEALTH AND ZOONOTIC RISK

In poultry, ACTA1-associated myopathies may exacerbate the impact of viral and bacterial infections. For instance, during highly pathogenic avian influenza outbreaks, birds with ACTA1 mutations are more susceptible to muscle failure, metabolic imbalance, and reduced activity, increasing the likelihood of morbidity and mortality [89–91].

Moreover, these mutations may impair intestinal motility, facilitating prolonged colonization by pathogens such as Campylobacter and Salmonella [92–97]. Dys-functional α-actin affects cytoskeletal dynamics in intestinal epithelial cells, weakening mucosal barriers and delaying peristalsis, which compromises digestive health and immune defense in infected birds.

## DIAGNOSTIC APPROACHES FOR ACTA1-RELATED MYOPATHIES

Diagnosis of ACTA1-related myopathies begins with a clinical assessment, focusing on muscle weakness, stiffness, or delayed motor development [98]. Definitive diagnosis is achieved through molecular testing, including gene expression profiling and DNA sequencing to identify ACTA1 variants [99].

Histopathological analysis of muscle biopsies can further reveal structural anomalies, such as fiber degeneration or protein accumulation [100]. Whole-genome sequencing techniques offer high-resolution insights into mutational hotspots and facilitate early detection, allowing for better-informed clinical decisions and disease management strategies [101].

## CURRENT AND FUTURE THERAPEUTIC STRATEGIES

At present, there is no specific cure for ACTA1-related myopathies, and available treatments focus on symptomatic relief, such as physical therapy and nutritional support [102, 103]. However, emerging molecular approaches, including gene therapy, RNA-based treatments, and small molecule stabilizers, show promise in correcting or compensating for defective ACTA1 expression [104].

With further research and clinical validation, precision medicine targeting ACTA1 dysfunction may become a feasible avenue to improve muscle function and disease outcomes in affected livestock and poultry.

## OVERVIEW OF ACTA1 FUNCTION IN SKELETAL MUSCLE DEVELOPMENT

The *ACTA1* gene, encoding skeletal α-actin, plays a critical role in sarcomere structure and skeletal muscle differentiation [77]. Proper expression of ACTA1 is tightly regulated to ensure optimal muscle development and function. As a core component of thin filaments, ACTA1 facilitates muscle contraction through its interaction with myosin and other contractile proteins [61, 105]. Any disturbance in its expression or structure may result in impaired sarcomeric integrity and muscular diseases, including congenital myopathies [87].

## TRANSCRIPTIONAL REGULATION OF ACTA1 EXPRESSION

Several muscle-specific transcription factors govern ACTA1 transcription during myogenesis, including MyoD, Myf5, and MEF2 [13, 55]. These transcription factors bind to the cis-regulatory regions in the ACTA1 promoter, triggering gene activation during the transition of myoblasts into myotubes [59, 71]. MEF2 coordinates extracellular signals with gene transcription to maintain developmental progression [66]. This cooperative network ensures spatiotemporal precision in ACTA1 expression throughout muscle growth stages [88].

## SIGNALING PATHWAYS INFLUENCING ACTA1 ACTIVATION

Beyond transcriptional factors, cellular signaling pathways such as Wnt/β-catenin, TGF-β, and Notch contribute to ACTA1 regulation [52, 92]. Wnt signaling upregulates MyoD, which indirectly stimulates ACTA1 expression during differentiation [106]. Conversely, TGF-β signaling inhibits MyoD activity, reducing ACTA1 expression and maintaining a balance between muscle proliferation and differentiation [107–109]. Disruptions in these pathways can impair ACTA1 synthesis, potentially contributing to developmental muscle abnormalities [47].

## EPIGENETIC REGULATION OF ACTA1 TRANSCRIPTION

Epigenetic modifications, including DNA methylation and histone acetylation, further fine-tune ACTA1 expression during muscle development [99]. Histone acetylation at the ACTA1 promoter enhances chromatin accessibility, allowing transcription factor binding and gene activation [110]. In contrast, DNA methylation may silence ACTA1 under specific physiological or pathological conditions [105]. These epigenetic layers integrate environmental cues and developmental signals, influencing long-term muscle gene expression and adaptability [56].

## ACTA1 DYSREGULATION AND NEUROMUSCULAR DISORDERS

Misregulation of ACTA1–either through mutations or altered transcriptional control–can result in various neuromuscular pathologies, especially congenital myopathies characterized by progressive muscle weakness and sarcomeric disorganization [35, 67, 81]. Mutations in ACTA1 impair α-actin’s ability to polymerize and interact with myosin, leading to defective muscle contraction and fiber degeneration [87, 111]. Understanding these molecular mechanisms is vital for early diagnosis and intervention in ACTA1-related diseases.

## THERAPEUTIC IMPLICATIONS AND FUTURE DIRECTIONS

Advancements in gene-editing technologies, particularly CRISPR-Cas9, have opened new avenues for correcting ACTA1 mutations at the genomic level [70]. In addition, stem cell-based approaches aim to restore functional muscle fibers by reintroducing genetically corrected cells. Targeting upstream transcriptional regulators and epigenetic modulators may offer new therapeutic strategies for patients with ACTA1-associated myopathies [58, 105]. Continued exploration of the regulatory network controlling ACTA1 will be crucial in developing precision therapies for muscle development disorders.

## INTRODUCTION TO ACTA1 GENE REGULATION IN MUSCLE DEVELOPMENT

The *ACTA1* gene, which encodes skeletal α-actin, plays a pivotal role in muscle development and function. Proper expression of this gene is critical for sarcomere assembly, muscle fiber differentiation, and contractile performance [112]. A complex interplay of transcriptional regulators and cis-regulatory elements orchestrates ACTA1 expression to ensure that skeletal α-actin is synthesized at the right time and in the correct cellular context during myogenesis [113, 114].

## ROLE OF MRFS

*ACTA1* gene expression is tightly controlled by MRFs; a family of transcriptional proteins that includes MyoD, MRF4, and Myogenin [115]. These factors are central to the myogenic program and function by binding to specific DNA regulatory motifs in the ACTA1 promoter, recruiting co-activators, and initiating transcription of skeletal α-actin [116, 117].

## MYOD: INITIATOR OF MYOGENIC DIFFERENTIATION AND ACTA1 EXPRESSION

MyoD is primarily responsible for initiating the transition from proliferation to differentiation in muscle progenitor cells. It acts as a master transcription factor that targets multiple muscle-specific genes, including ACTA1 [118]. During early muscle differentiation, MyoD binds to E-box motifs within the ACTA1 promoter, triggering its transcription and facilitating sarcomere formation in developing muscle fibers [119].

## MRF4: LONG-TERM MAINTENANCE OF ACTA1 IN MATURE MUSCLE

While MyoD dominates early stages of differentiation, MRF4 plays a more prominent role in maintaining ACTA1 expression in mature muscle cells [116]. It ensures the sustained synthesis of skeletal α-actin over the lifespan of the muscle cell, preserving muscle structure and contractile functionality [115]. Downregulation of MRF4 with aging may lead to muscle atrophy and dysfunction due to imbalanced contractile protein production [120].

## MYOGENIN: FINAL MATURATION AND STRUCTURAL INTEGRATION

Myogenin functions in the late stages of myogenic differentiation, coordinating the maturation of muscle fibers and the upregulation of ACTA1 [77]. It promotes sarcomeric assembly by not only enhancing ACTA1 transcription but also activating genes that encode structural proteins such as tropomyosin and desmin, which stabilize the cytoskeleton [42, 55]. Myogenin deficiency or dysfunction can impair sarcomere formation and lead to muscle development abnormalities or myopathies [19].

## MRF DYSFUNCTION AND ITS CONSEQUENCES IN MUSCLE PATHOLOGY

Disruption in the expression or function of MRFs can result in insufficient ACTA1 expression, leading to impaired sarcomere formation and muscle degeneration [61, 116]. Genetic mutations or dysregulation of MyoD, MRF4, or Myogenin may give rise to congenital myopathies and muscular dystrophies, where muscle fibers lose their ability to form or function effectively [33, 61, 121].

## REGULATORY ELEMENT INTERACTIONS AND ENHANCER DEPENDENCY

The precise control of ACTA1 expression relies on effective interactions between muscle-specific transcription factors and enhancer elements located within the gene’s regulatory regions [71]. Failure of these interactions, due to enhancer inactivation or mutation, may significantly downregulate ACTA1 transcription, compromising muscle fiber development and contractility [65].

## OVERVIEW OF MUSCLE FIBER TYPES AND ACTA1 INVOLVEMENT

Skeletal muscle fibers are broadly classified into Type I (slow-twitch) and Type II (fast-twitch) fibers. Type I fibers exhibit slower contraction but higher fatigue resistance, whereas Type II fibers contract rapidly and generate greater force, though they fatigue more easily [122, 123]. The expression of the *ACTA1* gene, which encodes skeletal α-actin, plays a critical role in determining the composition and functionality of these fiber types [43].

## ACTA1 EXPRESSION AND MUSCLE FIBER TRANSITION

**Figure 3:**
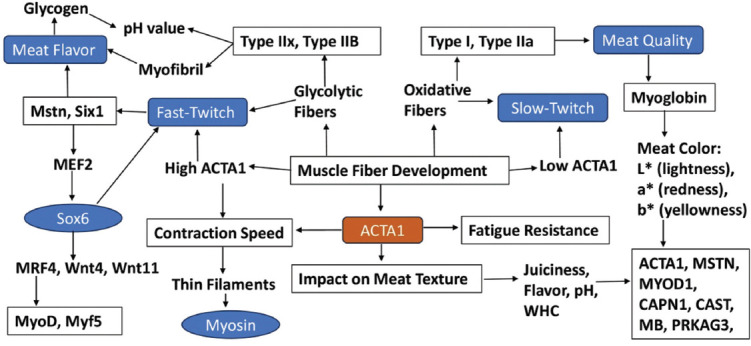
Comparison of ACTA1 in muscle fiber development between fast- and slow-twitch [Source: The figure was prepared by Siti Rani Ayuti].

Changes in ACTA1 expression levels influence the distribution and characteristics of muscle fibers, thereby affecting both meat quality and physical performance in livestock [72]. Elevated ACTA1 expression is associated with the development of larger and more robust Type II fibers, promoting greater muscle mass and leaner meat production [124]. Meanwhile, ACTA1 also contributes to fiber stability and contractile efficiency, supporting the functional maturation of muscle tissue [18, 54]. Figure 3 is an illustration of the comparison of ACTA1 in muscle fiber development between fast- and slow-twitch.

## SPECIES-SPECIFIC IMPLICATIONS FOR MEAT QUALITY

ACTA1 plays a vital role across diverse livestock species, including cattle, pigs, and poultry. In cattle, increased ACTA1 expression can promote Type I fiber formation, resulting in meat with softer texture and reduced intramuscular fat content [122]. In transgenic pigs, ACTA1 upregulation leads to hypertrophied muscles, yielding denser and higher-quality meat [65]. In poultry, targeted ACTA1 expression improves fiber uniformity and contractile integrity, contributing to more desirable meat characteristics [61, 64].

## GENETIC REGULATION OF MUSCLE FIBER COMPOSITION

The manipulation of *ACTA1* gene expression allows for selective enhancement of fiber type proportions, offering the potential to produce tailored meat textures to meet market demands. A higher proportion of Type I fibers typically produces more tender and juicy meat due to increased fat marbling, whereas a predominance of Type II fibers yields leaner, firmer meat with enhanced protein content [77, 80, 122, 123]. As a result, genetic selection strategies targeting ACTA1 may support consumer-oriented meat quality improvements [68, 124].

## MECHANICAL SIGNALING AND ACTA1 REGULATION

Mechanical stress during muscle contraction is one of the key stimuli regulating ACTA1 expression [125]. Muscle mechanoreceptors detect physical strain during exercise or weight-bearing activity, activating signaling cascades that upregulate *ACTA1* gene transcription [126]. This contributes to fiber hypertrophy and improved muscle function, directly influencing meat yield, and structural properties [127].

## EXERCISE, MUSCLE ADAPTATION, AND MEAT YIELD

Physical activity or environmental enrichment that promotes regular muscle use can increase ACTA1 expression through mechanotransduction pathways. This process supports fiber strengthening and endurance, which translates into higher meat quality through more uniform muscle development and improved texture and resilience [128, 129]. Understanding this mechanical-to-molecular link is essential for developing management practices and breeding programs that optimize ACTA1-related outcomes in livestock.

## HORMONAL AND GROWTH FACTOR INFLUENCE ON ACTA1 EXPRESSION

Hormones and growth factors play a crucial regulatory role in the expression of the *ACTA1* gene and the development of muscle fibers. Growth hormone and insulin-like growth factor 1 (IGF-1) have been shown to stimulate ACTA1 production in skeletal muscle, enhancing both hypertrophy and regeneration [130, 131]. For instance, IGF-1 promotes satellite cell proliferation and differentiation, ultimately contributing to muscle mass accumulation [132]. Similarly, testosterone enhances ACTA1-mediated muscle growth, supporting improvements in muscle yield and meat quality [56]. These hormonal effects can be exploited in feeding strategies or exercise protocols to fine-tune ACTA1 activity and thus muscle fiber development [11, 13].

## ACTA1 AND MUSCLE FIBER TYPING IN LIVESTOCK

The regulation of ACTA1 is a key determinant in the differentiation of Type I and Type II muscle fibers, which directly influences meat quality parameters such as tenderness, flavor, and marbling [133, 134]. Type I fibers are mitochondria-rich, fatigue-resistant, and associated with greater intramuscular fat, while Type II fibers are force-generating but fatigue-prone [133]. ACTA1 regulation modulates the balance and composition of these fiber types, providing opportunities to enhance meat characteristics through breeding and biotechnology [66, 88].

## TRANSCRIPTIONAL REGULATORS GOVERNING FIBER DIFFERENTIATION

Key transcription factors, MyoD, MEF2, and peroxisome proliferator-activated receptor gamma coactivator 1-alpha (PGC-1α), govern ACTA1 expression and are intimately involved in muscle fiber type specification [80, 134]. MEF2 and PGC-1α particularly support Type I fiber development, which is linked to tender meat with higher fat marbling [40]. Conversely, MyoD-driven expression supports Type II fiber development, favoring muscularity and meat density. The modulation of these transcription factors through exercise, diet, or epigenetic regulation can significantly alter meat properties in commercial livestock [44, 67].

## MARKET-ORIENTED MUSCLE FIBER MODULATION

Efficient regulation of ACTA1 and its downstream pathways provides a tool for tailoring muscle composition to market demands. Type I-dominant profiles are preferred for succulent and marbled meat, while Type II dominance supports lean, protein-rich meat suited for processed products [52, 134]. By modifying ACTA1 expression, producers can target-specific consumer preferences and improve market segmentation [19].

## GENETIC DIVERSITY AND ACTA1 POLYMORPHISMS

Genetic polymorphisms within the *ACTA1* gene influence fiber-type distribution, thereby affecting the tenderness and structure of meat across species. In cattle, these variations may determine the proportion of Type I vs. Type II fibers, affecting meat texture [134, 135]. Selective breeding based on ACTA1 variants enables the production of livestock lines with desirable muscle phenotypes, for example, softer meat from Type I-dominant animals or leaner, denser meat from Type II-biased genotypes [133].

In sheep, specific ACTA1 polymorphisms have been linked to a preference for Type I fiber formation, enhancing intramuscular fat content and tenderness [82,136]. Other variants may favor Type II development, supporting muscle hypertrophy but sometimes compromising meat softness [137, 138]. Thus, understanding and utilizing ACTA1 genetic variation is a powerful strategy for improving meat quality and productivity in livestock.

## OVERVIEW OF ACTA1 POLYMORPHISMS IN MUSCLE PHYSIOLOGY

Polymorphisms in the *ACTA1* gene, which encodes skeletal α-actin, significantly influence muscle fiber development, structure, and performance in livestock [55]. These genetic variations can modify the structure and functionality of the actin protein, a vital component of thin filaments responsible for muscle contraction and maintaining cellular architecture [40]. Alterations in the ACTA1 sequence may disrupt normal fiber development, thereby contributing to differences in muscle mass, strength, and endurance across individuals and species [12]. Importantly, these variations can directly influence the organoleptic qualities of meat, such as tenderness, flavor, and intramuscular fat content [11].

## POLYMORPHISMS AND MUSCLE FIBER DIFFERENTIATION

The role of ACTA1 polymorphisms is especially pronounced during myogenic differentiation, a critical phase in the formation of functional muscle fibers [32]. Sequence variations can affect actin expression levels or binding interactions, which in turn influence how satellite cells differentiate into type I (slow-twitch) or type II (fast-twitch) fibers [37]. Some polymorphisms enhance the proliferation or hypertrophy of specific muscle fiber types, resulting in larger muscles with improved structural properties, ultimately enhancing meat yield and quality [76, 82].

## IMPACT ON FIBER-TYPE COMPOSITION AND MEAT TEXTURE

ACTA1 polymorphisms can shift the balance between type I and type II fibers, a ratio critical for determining meat texture, tenderness, and metabolic profile [133]. Type I fibers confer resilience and marbling, while type II fibers contribute to density and rapid force generation [12, 18]. Genetic variants influencing ACTA1 expression can tip this balance, enabling producers to tailor fiber composition based on desired meat characteristics. Such polymorphisms offer a valuable tool for precision breeding aimed at enhancing consumer-preferred meat traits.

## ADAPTATION TO ENVIRONMENTAL AND PHYSIOLOGICAL STRESS

In addition to influencing baseline muscle physiology, ACTA1 polymorphisms also contribute to muscle adaptability under environmental or physiological stressors [37]. Certain alleles may enhance muscle regeneration, oxidative stress resistance, or thermal tolerance, which are crucial traits for livestock raised in suboptimal or fluctuating environments [59]. These polymorphisms can improve muscle healing, reduce fatigue, and support performance in endurance-based activities [19].

For example, variants that upregulate ACTA1 expression may favor the development of type II fibers, optimizing livestock for rapid growth and high protein output [133]. Conversely, polymorphisms promoting type I fiber dominance may improve muscle endurance and resistance to fatigue, making them ideal for animals in free-range or labor-intensive systems [134].

## APPLICATIONS IN BREEDING FOR ENHANCED MEAT PRODUCTION

Harnessing ACTA1 polymorphisms through genetic selection strategies offers promising avenues for improving muscle quality, productivity, and adaptability in commercial livestock [9]. Molecular breeding programs can incorporate genotype screening to identify favorable ACTA1 variants, enabling the selection of animals with optimal fiber composition and performance traits. These insights can be integrated into MAS or genome editing approaches, laying the groundwork for more efficient, targeted improvements in meat production systems.

## ENHANCING ACTA1 EXPRESSION THROUGH GENETIC SELECTION

The *ACTA1* gene, essential for skeletal muscle fiber development and meat quality, is a critical target in modern livestock breeding programs [138]. Optimizing ACTA1 expression can yield meat with greater muscle mass, improved texture, and more efficient production metrics [11]. Genetic selection technologies allow breeders to leverage natural genetic variation in ACTA1 to select individuals with beneficial expression profiles [52]. One of the most effective strategies is marker-based selection, which identifies animals carrying alleles associated with enhanced ACTA1 expression [13, 66].

By detecting single-nucleotide polymorphisms linked to ACTA1 activity, breeders can select for traits aligned with production goals, such as higher proportions of type I muscle fibers for tender meat with increased marbling, or type II fibers for leaner, denser cuts [133, 134]. These molecular tools reduce the need for labor-intensive phenotypic screening, thus accelerating genetic improvement in livestock [12]. Marker-based selection has already shown substantial results in beef and swine production systems, where ACTA1 regulation plays a key role in meat quality outcomes [72].

## SUSTAINABLE MEAT PRODUCTION THROUGH ACTA1 OPTIMIZATION

Strategic breeding to upregulate ACTA1 can significantly improve meat yield, feed efficiency, and environmental sustainability in the livestock sector [135]. Enhanced ACTA1 expression increases muscle development, allowing animals to convert feed more efficiently into lean tissue, thereby lowering production costs and reducing environmental impact [136, 137]. Furthermore, producing premium meat with superior texture and fat distribution opens new marketing opportunities, particularly in high-value consumer segments [12, 19].

## GENOME-WIDE ASSOCIATION STUDIES (GWAS): UNCOVERING GENETIC MARKERS FOR ACTA1 PERFORMANCE

GWAS offer a powerful approach for identifying genetic variants associated with ACTA1 regulation across large livestock populations [139]. Through high-throughput genotyping and phenotypic mapping, GWAS enables the discovery of alleles influencing feed conversion, muscle fiber type, and overall meat quality [140, 141]. These insights allow breeders to trace polygenic traits back to specific loci, facilitating targeted interventions to improve muscle performance traits regulated by ACTA1 [142, 143].

## INTEGRATING GWAS, MAS, AND CRISPR FOR MAXIMUM EFFICIENCY

The integration of *CRISPR* gene editing, GWAS-derived genetic insights, and MAS offers a comprehensive framework for optimizing ACTA1 expression in livestock breeding [144, 145].

- CRISPR-Cas9 technology allows for precise genetic enhancement of ACTA1 function by directly editing regulatory or coding sequences [146].
- MAS enables the selection of individuals with desirable ACTA1-linked genotypes without requiring full-genome sequencing [147].
- GWAS provides population-level insights to guide polygenic trait selection and long-term breeding program development [148].

This integrated strategy enhances genetic accuracy, breeding speed, and meat production efficiency, ultimately supporting a sustainable and profitable livestock industry.

## CONCLUSION

This review comprehensively highlights the critical role of the *ACTA1* gene in regulating skeletal muscle development, fiber composition, and meat quality across various livestock species. ACTA1 influences the formation of both type I and type II muscle fibers, directly affecting meat tenderness, intramuscular fat content, and overall carcass composition. Its expression is governed by a complex network of transcription factors such as MyoD, Myogenin, and MEF2, signaling pathways, such as Wnt, Notch, and TGF-β, and epigenetic modifications including DNA methylation and histone acetylation. These interactions underscore the gene’s integrative role in myogenesis, muscle function, and phenotypic variation among livestock breeds.

Optimizing ACTA1 expression through genetic selection, nutritional modulation, and modern biotechnology, including *CRISPR-Cas9* gene editing and MAS, offers a promising avenue to enhance muscle yield, meat quality, and feed efficiency in livestock. These molecular and genetic interventions enable producers to selectively breed animals with superior muscle traits, reduced fat deposition, and improved meat texture, meeting both economic and consumer demands. Moreover, ACTA1’s role in regulating muscle fiber composition aligns with industry goals for producing high-value meat products and sustainable animal production systems.

A key strength of this review is its multidisciplinary integration of molecular biology, genetics, epigenetics, and practical animal husbandry. It provides a bridge between foundational gene regulation mechanisms and applied outcomes in livestock performance. However, the review is constrained by a limited number of functional studies specifically focusing on ACTA1 across a wide range of livestock species, particularly under varying environmental and physiological stress conditions. In addition, long-term field data linking ACTA1 polymorphisms to lifetime meat yield and quality are lacking.

Future research should aim to elucidate ACTA1’s species-specific regulatory networks, identify functional polymorphisms associated with environmental adaptability, and explore the potential of nutrigenomic interventions to fine-tune gene expression. Integrating transcriptomic, proteomic, and phenotypic datasets will be essential for designing precision breeding programs that target ACTA1-related traits. With growing global demand for high-quality protein, strategic and ethical enhancement of ACTA1 expression holds the potential to revolutionize meat production, improve economic returns, and support food security. Through sustained innovation and responsible application, ACTA1-based approaches can shape the future of precision livestock farming.

## DATA AVAILABILITY

All references are open access, so data can be obtained from the online websites.

## AUTHORS’ CONTRIBUTIONS

SRA, SS, and EJK: Conducted a literature search and drafted the manuscript. ML, SHW, MAA, and WPL: Revised and edited the manuscript. ZNAR, ARK, and MA: Drafted and revised the manuscript. MZR, RR, and MD: Edited the references. All authors have read and approved the final manuscript.
